# Identification of hepatitis B and C screening and patient management guidelines and availability of training for chronic viral hepatitis among health professionals in six European countries: results of a semi-quantitative survey

**DOI:** 10.1186/s12879-015-1104-8

**Published:** 2015-08-19

**Authors:** Angela Bechini, Abby Falla, Amena Ahmad, Irene Veldhuijzen, Sara Boccalini, Barbara Porchia, Miriam Levi

**Affiliations:** Department of Health Sciences, University of Florence, Florence, Italy; Department of Public Health, Erasmus MC, University Medical Center Rotterdam, Rotterdam, The Netherlands; Department of Health Sciences, Hamburg University of Applied Sciences, Hamburg, Germany; Division of Infectious Disease Control, Public Health Service Rotterdam-Rijnmond, Rotterdam, The Netherlands

## Abstract

**Background:**

As part of the EU funded project “HEPscreen”, the aim of this study is to identify hepatitis B and C screening and patient management guidelines, to assess the awareness of these among health professionals (HPs) and to explore the availability of hepatitis B/C training programmes for HPs in Germany, Italy, the Netherlands, the UK, Spain and Hungary.

**Methods:**

A comprehensive literature search through the main scientific databases was performed to retrieve guidelines, following which an online survey was developed and sent to HPs in six areas of health care, including public health, to verify whether HPs are aware of these guidelines, to retrieve additional guidelines and to find out whether specific professional training is available.

**Results:**

Twelve national guidelines were identified through the literature search. Of the 268 respondents, 80 % were aware of hepatitis B guidelines and 73 % were aware of hepatitis C guidelines in their country. The national guidelines identified through the literature search were mentioned by 1/3 of HPs in the UK and Germany, 13 % of HPs in the Netherlands, 14 % in Italy and 4 % in Spain. An additional 41 hepatitis B/C related guidance documents were retrieved through the online survey: 15 in the UK, seven in Hungary, six in Italy, five in the Netherlands, four in Germany and four in Spain. Availability of training programmes to improve skills and knowledge in viral hepatitis was most often reported in the Netherlands, with 82 % indicating availability and just 10 % indicating no availability, and least commonly in Italy, with 42 % indicating yes but 40 % indicating no. Availability was also reported by the majority in the UK, Hungary and Spain, while in Germany the majority selected unsure.

**Conclusions:**

Results suggest that the scientific databases are not the most important information source of best clinical practice for many HPs. Implementation of best practices requires that guidelines are specifically designed and actively promoted among those who are to follow them. Training can disseminate these best practice recommendations and raise awareness of guidelines. It is therefore encouraging that diverse training about hepatitis B/C is available to the different professional groups.

**Electronic supplementary material:**

The online version of this article (doi:10.1186/s12879-015-1104-8) contains supplementary material, which is available to authorized users.

## Background

Chronic hepatitis B and C are leading causes of liver cancer and are both important public health issues in Europe. In the European Union (EU), some segments of the population, such as migrants from areas where HBV or HCV are endemic and people who inject drugs (PWID), are disproportionately affected by these diseases [1–4]. The prevalence in the general population varies from 0.4 to 5.2 % for anti-HCV and from 0.1 to 5.6 % for HBsAg [5]. However, also due to the largely silent nature of these infections, reliable epidemiological data in Europe are lacking both on HBV and HCV [6, 7] and it has been estimated that up to 90 % of infected individuals are undiagnosed [8]. Therefore, despite the existence of effective antiviral treatment that slows disease progression and prevents the development of cirrhosis and liver cancer, many patients who might benefit from treatment remain undetected [9, 10]. Studies also allude to ineffective counselling and referral of diagnosed patients, as well as to the failure of chronically infected patients to reach secondary care, leading to eligible viral hepatitis patients being under-treated [11–16].

Informing health professionals (HPs) of evidence-based recommendations on the prevention of hepatitis B and C, the targeted screening of at-risk individuals, and the diagnostics and clinical management of patients with chronic viral hepatitis, is crucial to obtain the best possible health outcomes. The provision of comprehensive high quality guidelines, as well as advanced training programmes to improve the skills and knowledge of HPs on viral hepatitis management, are ways to achieve this purpose. Numerous studies, however, demonstrate little familiarity or low compliance of HPs with guidelines summarising the best available evidence in their specialties [17–21]. National and European hepatitis B and C management guidelines exist, however little is known about the extent to which HPs who are to actually implement them are aware of their existence. Similarly, little is known about the availability of in-service training on chronic viral hepatitis prevention, diagnosis, management and treatment.

This study, conducted as part of EU HEPscreen, a project co-funded by the EU Health Programme (www.hepscreen.eu), has four specific aims. First, to provide an overview of published hepatitis B and C clinical practice guidelines available in Europe and in particular in six EU countries with large migrant communities and representation of migrant health and patient platform, i.e. the UK, Germany, the Netherlands, Hungary, Italy and Spain. Second, to assess the awareness of guidelines among HPs working in these six countries in six fields: public health, antenatal care, primary care, care for asylum seekers/refugees, sexual health, and gastroenterology/hepatology. Third, to measure the availability of viral hepatitis specific training programmes for HPs in these fields. Finally, to investigate HPs’ opinion on the existence of barriers such as limited guidance available to primary health care professionals about onward referral, counselling and patient management of hepatitis B/C patients and low training uptake among professionals as explanations of why hepatitis B/C cases do not reach specialized care for further investigation and treatment.

## Methods

First, a comprehensive literature search was conducted to retrieve published national and European hepatitis B and C clinical guidelines. A modified version of the “PICO” method [22] was applied, using a search syntax comprising of four categories: (1) *Population:* “general population” OR migrants OR “sex workers” OR “Intravenous drug users” OR IDUs; (2) *Disease:* “hepatitis B” OR “hepatitis C”; (3) *Intervention:* screening OR counselling OR referral OR treatment OR therapies OR “clinical management”; (4) *Setting:* Germany OR Hungary OR Italy OR Spain OR the Netherlands OR the UK OR Europe. These four categories were combined with “AND” to build the final syntax. MEDLINE, EMBASE and the Cochrane Library databases were searched. In addition, websites of the National Institute for Health and Clinical Excellence, the Scottish Intercollegiate Guidelines Network, the Italian National Guidelines System, the European Association for the Study of the Liver (EASL), the European Centre for Disease Prevention and Control (ECDC), the Robert‐Koch‐Institute, the World Health Organization, the World Hepatitis Alliance, the European Liver Patients Association and the Italian Liver Patient Association, were searched. The literature search encompassed guidelines published between January 2000 to March 2012 in English, Spanish, Italian, Dutch, Hungarian or German and it was conducted between November 2011 and February 2012. Titles, abstracts and full-texts of relevant documents were screened by two reviewers, independently. Disagreements about eligibility were resolved through discussion. A list of all guidelines retrieved was developed and categorised by country and type of hepatitis.

To identify further professional guidelines missed by the literature search and to assess awareness about existing guidelines among different groups of HPs involved in screening for or caring for chronic viral hepatitis, six semi-quantitative online surveys were developed. The surveys were sent to HPs in their respective field i.e. to public health professionals (PHPs); to general practitioners (GPs); to sexual health service providers (SHS) and/or genitourinary medicine (GUM) specialists; to antenatal care (ANC) providers; to asylum seeker care (ASC) providers and to specialists (SP) in the field of gastroenterology/hepatology and infectious diseases. Each survey was pilot tested, translated into the national language of the respective study countries, uploaded into the open source online software package LimeSurvey, and sent by e-mail to HPs in the six areas, who were identified by board membership of clinical associations, professional networks, ECDC focal points, scientific literature and other means. Rather than to reach a large representative sample of practising clinicians, the aim in each professional group was to reach 5–10 HPs deemed able to reflect on the practice within their specialism in general. Data were collected between July and September 2012. Respondents agreed to participate by answering the questionnaire.

We measured whether official national guidelines were available, both general guidelines (the question was included in all six surveys), with a request to provide the title and publisher in a text field in case of a positive response, and guidelines specifically developed for professionals in their field. We also asked public health specialists whether specific guidelines for migrants from endemic areas exist. The responses were exported into SPSS 19.2 and a descriptive analysis was performed to evaluate which hepatitis B/C guidelines are known to HPs, how many professionals mentioned the main guidelines identified through the literature search and how many additional guidelines were retrieved. In all surveys, except the survey aimed at public health professionals, a question about the provision of hepatitis B/C-related training for HPs in their respective medical specialties was also included. The identification of general hepatitis guidelines by respondents was analysed in connection with their opinion on the existence of professional training to improve knowledge and skills about viral hepatitis. Finally, all professional groups were asked to indicate on a five-point Likert scale, from “strongly agree” to “strongly disagree", to which extent they agree that the given statements are explanations of why hepatitis B/C cases do not reach specialized health care for further management: i) There is limited guidance available to primary health care professionals about onward referral, counselling and patient management of hepatitis B/C patients; ii) Although training on viral hepatitis management is available for health care providers, uptake is generally low among professionals. The study complied with the Helsinki Declaration [23].

## Results

### Literature search results: clinical practice guidelines

The literature search retrieved eight international guidelines: two from EASL [24, 25]; two from the Association for the Study of Liver Diseases [26, 27]; the “European Guideline for the Management of Hepatitis B and C Virus Infections” by the International Union against Sexually Transmitted Infections [28]; the “Best Practice in the Treatment of Chronic Hepatitis B” published by the European Viral Hepatitis Educational Initiative [29]; and two National Institute of Health Consensus Statements on the management of hepatitis C and B from the US [30, 31]. Twelve major national guidelines were retrieved: six in the UK [32–37], two in Italy [38, 39], two in Germany [40, 41] and two in the Netherlands [42, 43].

Since the date of the search (March 2012), several guidelines have been revised, mostly due to treatment advances. In such cases, the updated guidelines are cited as references.

### Survey results

We received a total of 268 responses to the survey, not evenly distributed across the six professional groups or across the six countries (Table 1).

**Table 1:** Number of health professionals completing the questionnaire by country and by survey

Survey (% of health professionals for each survey by country)	Country						Total
	UK	DE	NL	HU	IT	ES	
Public health	9 (20 %)	13 (29 %)	7 (16 %)	2 (4 %)	8 (18 %)	6 (13 %)	45 (100 %)
Antenatal care	8 (10 %)	33 (40 %)	6 (7 %)	4 (5 %)	23 (28 %)	8 (10 %)	82 (100 %)
GP	8 (21 %)	4 (11 %)	9 (24 %)	1 (3 %)	14 (37 %)	2 (5 %)	38 (100 %)
Care for asylum seekers	4 (22 %)	3 (17 %)	4 (22 %)	3 (17 %)	3 (17 %)	1 (6 %)	18 (100 %)
SHS	9 (35 %)	4 (15 %)	7 (27 %)	3 (11 %)	1 (4 %)	2 (8 %)	26 (100 %)
Specialist care	9 (15 %)	7 (12 %)	22 (37 %)	8 (14 %)	9 (15 %)	4 (7 %)	59 (100 %)
Total	47 (18 %)	64 (24 %)	55 (21 %)	21 (8 %)	58 (22 %)	23 (9 %)	268 (100 %)

### Identification of HBV and HCV guidelines through the online survey

National or international guidelines already identified through the literature search were mentioned by one third of respondents in the UK and Germany, and only by a minority of HPs in Italy (14 %) and the Netherlands (13 %) and by just 4 % in Spain. An additional 41 hepatitis B/C-related national guidance documents were identified by HPs through the online survey: 15 in the UK, seven in Hungary, six in Italy, five in the Netherlands, four in Germany and four in Spain. Additional file 1 Table S1 compiles all hepatitis B/C related guidelines and guidance documents retrieved through the literature search and/or identified by HPs.

### National general HBV and HCV screening and management guidelines

The existence of official national general hepatitis B or C screening and patient management guidelines in the study countries, was reported by 61 % and 56 %, respectively (Table 2). Among these, only about 40 % provided the title and publisher of the guideline. Around two thirds to three quarters of HPs in the Netherlands and in Hungary reported availability of general guidelines compared to just over half in the UK and Italy. Conversely, the majority of HPs in Spain and, for hepatitis C guidelines, in Germany, reported uncertainty or that no general guideline is available.

**Table 2:** Health professionals reporting the existence of national general hepatitis B and C guidelines in their country

Hepatitis B guidelines	UK (*n* = 47)	DE (*n* = 64)	NL (*n* = 55)	HU (*n* = 21)	IT (*n* = 58)	ES (*n* = 23)	Total (*n* = 268)
Proportion of health professionals reporting the existence	57 %	56 %	78 %	67 %	57 %	43 %	61 %
Provided name and publisher^a^	41 %	31 %	44 %	36 %	45 %	50 %	40 %
Hepatitis C guidelines	UK (*n* = 47)	DE (*n* = 64)	NL (*n* = 55)	HU (*n* = 21)	IT (*n* = 58)	ES (*n* = 23)	Total (*n* = 268)
Proportion of health professionals reporting the existence	60 %	47 %	67 %	67 %	57 %	39 %	56 %
Provided name and publisher^b^	39 %	37 %	51 %	29 %	45 %	44 %	42 %

^a^Percent of the respondents who reported the existence of general hepatitis B guidelines

^b^Percent of the respondents who reported the existence of general hepatitis C guidelines

### Professional group-specific national HBV and HCV guidelines

Overall 80 % (*n* = 215) of respondents are aware of hepatitis B guidelines and 73 % (*n* = 196) of hepatitis C guidelines in their country. Among the 45 PHPs, around two thirds mentioned the existence of general Hepatitis B and hepatitis C guidelines. Among the 38 GPs, 29 % mentioned specific HBV guidelines for GPs and 21 % the existence of HCV guidelines for GPs. Interestingly, among PHPs, 47 % mentioned the existence of HBV guidelines specifically for GPs and 40 % the existence of GP-specific HCV guidelines. Of the 82 ANC experts, 52 % mentioned HBV and 26 % HCV guidelines for antenatal services. Among the 59 secondary care specialists, 61 % mentioned HBV guidelines and 56 % mentioned HCV guidelines. None of the 18 ASC experts identified the existence of HBV/HCV guidelines for the care of asylum seekers. Just 22 % (for HBV) and 13 % (for HCV) of PHPs reported the existence of specific guidelines for migrants from endemic areas. Detailed results are displayed in Table 3.

**Table 3:** Health professionals identifying hepatitis general or specific guidelines by professional group and by country

HBV GUIDELINES	UK	DE	NL	IT	ES	HU	Total
General Hepatitis B guidelines							
Public health professionals	56 %	77 %	71 %	50 %	67 %	100 %	67 %
Antenatal care experts	50 %	33 %	67 %	44 %	13 %	50 %	39 %
General Practitioners	38 %	50 %	78 %	71 %	100 %	100 %	66 %
Asylum seekers Experts	100 %	100 %	50 %	100 %	0 %	100 %	83 %
SHS Experts	56 %	75 %	86 %	100 %	0 %	67 %	65 %
Specialists	67 %	100 %	86 %	56 %	75 %	50 %	75 %
GP guidelines							
Public health professionals	67 %	31 %	100 %	25 %	33 %	0 %	47 %
General Practitioners	13 %	0 %	78 %	21 %	0 %	0 %	29 %
Antenatal guidelines							
Public health professionals	78 %	8 %	71 %	25 %	67 %	50 %	44 %
Antenatal care Experts	75 %	40 %	67 %	39 %	88 %	100 %	52 %
Asylum seekers guidelines							
Public health professionals	33 %	0 %	14 %	13 %	0 %	0 %	11 %
Asylum seekers Experts	0 %	0 %	0 %	0 %	0 %	0 %	0 %
Specialists guidelines							
Public health professionals	78 %	38 %	57 %	25 %	33 %	50 %	47 %
Specialists	44 %	43 %	86 %	33 %	75 %	50 %	61 %
Specific migrant care guidelines							
Public health professionals reporting the existence of specific migrant care guidelines	33 %	0 %	14 %	25 %	67 %	0 %	22 %
HCV GUIDELINES	UK	DE	NL	IT	ES	HU	Total
General Hepatitis C guidelines							
Public health professionals	56 %	77 %	57 %	50 %	67 %	100 %	64 %
Antenatal care experts	38 %	18 %	67 %	44 %	25 %	25 %	32 %
General Practitioners	38 %	50 %	67 %	71 %	50 %	100 %	61 %
Asylum seekers Experts	100 %	100 %	50 %	100 %	0 %	100 %	83 %
SHS Experts	67 %	75 %	71 %	100 %	0 %	67 %	65 %
Specialists	78 %	86 %	73 %	56 %	50 %	63 %	70 %
GP guidelines							
Public health professionals	67 %	23 %	57 %	38 %	33 %	0 %	40 %
General Practitioners	0 %	0 %	56 %	21 %	0 %	0 %	21 %
Antenatal guidelines							
Public health professionals	33 %	0 %	14 %	25 %	0 %	0 %	13 %
Antenatal care experts	25 %	13 %	0 %	35 %	75 %	25 %	26 %
Asylum seekers guidelines							
Public health professionals	11 %	0 %	0 %	13 %	0 %	0 %	4 %
Asylum seekers Experts	0 %	0 %	0 %	0 %	0 %	0 %	0 %
Specialists guidelines							
Public health professionals	56 %	23 %	29 %	25 %	0 %	0 %	27 %
Specialists	44 %	43 %	73 %	44 %	50 %	50 %	56 %
Specific migrant guidelines							
Public health professionals reporting the existence of specific migrant care guidelines	33 %	0 %	0 %	13 %	33 %	0 %	13 %

### Reported availability of hepatitis B/C training for health care professionals

Table 4 shows the reported availability of training in the six countries. Among secondary care specialists, availability of training was reported by 84 % (although only by 44 % in Italy), among GPs by two thirds (although only by half of them in Germany and Spain and by 40 % in the UK) and by 55 % among SHS experts. Most HPs working in antenatal care indicated that training is not available or selected unsure. The majority opinion among those providing care to asylum seeker is that training is not available for professionals in their field.

**Table 4:** Availability of training to improve knowledge and skills in viral hepatitis in the six countries

UK	GP (*n* = 10)	Antenatal (*n* = 8)	Asylum (*n* = 4)	SHS (*n* = 10)	Specialist (*n* = 10)
Yes	40 %	50 %	25 %	70 %	100 %
No	10 %	13 %	75 %	0 %	0 %
Unsure	50 %	38 %	0 %	30 %	0 %
DE	GP (*n* = 4)	Antenatal (*n* = 36)	Asylum (*n* = 3)	SHS (*n* = 5)	Specialist (*n* = 9)
Yes	50 %	11 %	67 %	60 %	67 %
No	0 %	25 %	33 %	0 %	0 %
Unsure	50 %	64 %	0 %	40 %	33 %
NL	GP (*n* = 9)	Antenatal (*n* = 6)	Asylum (*n* = 4)	SHS (*n* = 8)	Specialist (*n* = 22)
Yes	89 %	33 %	75 %	63 %	100 %
No	0 %	50 %	25 %	13 %	0 %
Unsure	11 %	17 %	0 %	25 %	0 %
HU	GP (*n* = 1)	Antenatal (*n* = 4)	Asylum (*n* = 3)	SHS (*n* = 3)	Specialist (*n* = 10)
Yes	100 %	50 %	33 %	0 %	80 %
No	0 %	25 %	33 %	100 %	0 %
Unsure	0 %	25 %	33 %	0 %	20 %
IT	GP (*n* = 14)	Antenatal (*n* = 25)	Asylum (*n* = 3)	SHS (*n* = 1)	Specialist (*n* = 9)
Yes	79 %	28 %	0 %	0 %	44 %
No	7 %	52 %	100 %	100 %	33 %
Unsure	14 %	20 %	0 %	0 %	22 %
ES	GP (*n* = 2)	Antenatal (*n* = 8)	Asylum (*n* = 1)	SHS (*n* = 2)	Specialist (*n* = 4)
Yes	50 %	75 %	0 %	50 %	100 %
No	50 %	25 %	100 %	0 %	0 %
Unsure	0 %	0 %	0 %	50 %	0 %

### Guidelines and Training

Availability of general national guidelines was most commonly mentioned by HPs who reported the existence of professional training (Table 5). Over two thirds of those who indicated that professional training is available also indicated the existence of general guidelines for hepatitis B (69 %) and hepatitis C (64 %), whereas among those who indicated that training is not available, just 47 % for HBV and 42 % for HCV reported the existence of general national guidelines. Surprisingly, in Hungary, general hepatitis B guidelines were identified more often by HPs reporting a lack of, or uncertainty about, available training for professionals.

**Table 5:** Identification of general guidelines by health professionals in relation to the perceived availability of training

Health professionals reporting that training *is* available for professionals		UK	DE	NL	HU	IT	ES	Tot
	Health professionals mentioning hepatitis B guidelines	15/25 (60 %)	14/17 (82 %)	34/40 (85 %)	7/12 (58 %)	13/22 (59 %)	5/12 (42 %)	88/128 (69 %)
	Health professionals mentioning hepatitis C guidelines	15/25 (60 %)	12/17 (71 %)	29/40 (73 %)	8/12 (67 %)	13/22 (59 %)	5/12 (42 %)	82/128 (64 %)
Health professionals reporting that training is *not* available for professionals or who were *unsure*		UK	DE	NL	HU	IT	ES	Tot
	Health professionals mentioning hepatitis B guidelines	7/13 (54 %)	12/34 (35 %)	4/8 (50 %)	5/7 (71 %)	16/28 (57 %)	1/5 (20 %)	45/95 (47 %)
	Health professionals mentioning hepatitis C guidelines	8/13 (62 %)	8/34 (24 %)	4/8 (50 %)	4/7 (57 %)	16/28 (57 %)	0/5 (0 %)	40/95 (42 %)

Public health professionals’ responses were not taken into consideration, since the question on the availability of training was not asked in the survey aimed at public health professionals

### Perceived barriers to inadequate referral of hepatitis B/C cases

Limited guidance available to primary health care professionals about onward referral, counselling and patient management of hepatitis B/C patients was perceived as a reason of why hepatitis B/C patients do not reach specialized health care for further investigation and treatment according to nearly half of the respondents in Italy (43 % answered they “agree” or “strongly agree”), around a third of respondents in Spain, a quarter in the UK and in Germany and a fifth in the Netherlands, but not in Hungary (Table 6). Low training uptake among professionals as a possible explanation was reported by more than half of the respondents in Italy (54 % agreed or strongly agreed with such a statement), by 38 % of those in the UK, a third in the Netherlands, along with a sixth in Germany in Hungary and 9 % in Spain (Table 6).

**Table 6:** Health professionals’ opinion on the existence of barriers as explanations of why hepatitis B/C cases do not reach specialized health care (e.g. hepatologists) for further investigation and treatment. Results are presented by country

		UK (*n* = 47)	DE (*n* = 64)	NL (*n* = 55)	HU (*n* = 21)	IT (*n* = 58)	ES (*n* = 23)
There is limited guidance available to primary health care professionals about onward referral, counselling and patient management of hepatitis B/C patients.	Strongly disagree	11 %	8 %	2 %	33 %	7 %	17 %
	Disagree	32 %	31 %	58 %	29 %	34 %	39 %
	Neither agree or disagree	30 %	38 %	18 %	33 %	16 %	13 %
	Agree	21 %	20 %	18 %	5 %	40 %	30 %
	Strongly agree	6 %	3 %	4 %	0 %	3 %	0 %
Although training on viral hepatitis management is available for health care providers, uptake is generally low among professionals.	Strongly disagree	2 %	8 %	2 %	29 %	3 %	13 %
	Disagree	23 %	31 %	9 %	24 %	21 %	43 %
	Neither agree or disagree	36 %	45 %	56 %	33 %	22 %	35 %
	Agree	34 %	16 %	31 %	10 %	52 %	9 %
	Strongly Agree	4 %	0 %	2 %	5 %	2 %	0 %

In Additional file 2, results in Tables 2–4 and 6 are reported in absolute numbers.

## Discussion

It has been estimated that in Europe only a minority of hepatitis B or C cases are diagnosed, and that less than 20 % of infected individuals receive treatment [44]. The reasons for the low treatment rate include on one hand the largely silent nature of the disease, described accordingly as “the silent epidemic”, which often prevents patients to seek care until the disease has progressed to end-stage liver disease or hepatocellular carcinoma. On the other hand, patients’ lack of knowledge about the disease, language difficulties, lack of social support and lack of understanding of the healthcare system contribute to the exclusion from health care of migrants from endemic areas, despite current evidence showing that they account for one of the largest HBV- or HCV-infected group in Europe [45]. Legal, administrative, financial barriers and the stigmatization of certain at-risk groups, such as sex workers or PWID, also represent major obstacles to an effective clinical management of this condition [46, 47]. Models show that, with treatment at current levels, mortality related to HCV is expected to rise and to peak around 2030 [48]. Cohen et al. recently introduced the concept of “under-treatment” to refer to the disparity between the number of chronically infected individuals and the number of patients receiving treatment [12]. In order for chronic viral hepatitis-related morbidity and mortality to stop to rise in Europe, large increases in early detection and treatment of patients are urgently needed.

Our study set out to measure availability and awareness of two important means through which evidence-based recommendations can influence clinical practice and HPs’ action towards effective clinical management, i.e. guidelines and training. Availability of general hepatitis guidelines was most commonly reported by HPs for whom professional training is also available in their country. Encouragingly, we identified a total of 53 national hepatitis B or C guidelines and guidance documents, with examples across the six countries. However, just twelve of these were retrieved via the literature search. For Hungary, the search failed to retrieve any guidelines published in English, however seven guidelines, all in Hungarian, were retrieved via the online survey. Moreover, in accordance with prior studies [17–21], few HPs themselves identified guidelines published in the scientific literature. These findings suggest that the scientific databases are not the most important information source of best clinical practice for many HPs. Interestingly, more public health officials identified specific guidelines for GPs than GPs themselves. Limited guidance available was perceived as a reason for inadequate referral of patients according to sizeable proportions of HPs in all countries, with the exception of Hungary. More comprehensive guidelines, tailored to the needs of specific professional groups like GPs, sexual health or maternity services, may be an alternative to increase awareness and improve implementation of recommendations. Our findings also indicate that there is either scarcity or complete lack of guidance for HPs about screening practices and disease management of migrants and asylum seekers from endemic areas.

Results on the availability of specific hepatitis B/C training programmes suggest that, for many professionals, in the Netherlands, the UK, Hungary and Spain training is available. However there are differences between professional groups within countries. Results in the UK suggest that training is lacking or unknown for GPs and professionals in health care for asylum seekers. In Germany, training is similarly neither widespread nor well known across all professional groups. In Hungary, training is not widely available, especially for professionals working in the area of sexual health. In Italy, a lack of availability was reported by over half of the antenatal care providers and by all respondents to the asylum seeker and SHS survey; training seems to be more available to GPs. The low numbers of respondents among some professional groups in Hungary and Spain limit the generalizability of findings, although in Spain, training seems to be available for antenatal care providers and specialists.

That training is most commonly available to secondary care specialists is perhaps not surprising; what is more surprising is that less than half in Italy and only two thirds of specialists in Germany reported the availability of training. Given the role of the specialists and the rapidly advancing knowledge of viral hepatitis, especially the new treatment options for hepatitis C, this finding is particularly concerning. Results suggest that, except for Spain, training for antenatal care providers is rather limited, but especially so in Germany, Netherlands and Italy. The implications of this could be sub-optimal care and ineffective referral of pregnant women testing positive, as well as a lack of contact tracing. It would be particularly interesting to know how this lack of training has an impact on the care of hepatitis B positive pregnant women. Low training uptake as a possible explanation of why hepatitis B/C cases do not reach specialized health care was reported by relevant proportions of HPs in Italy, in the UK, and in the Netherlands.

The findings from our study highlighted that the awareness of screening and patient management guidelines and in-service training courses among HPs are presently insufficient. Improving results, as supported by key stakeholders [49], would imply a strong involvement of national health authorities with the implementation of specific national action plans, an effective disease surveillance to develop effective policies and the establishment of specialized centers. In this respect, the undeniable success of the experiences developed in Scotland and in France provide a working model for other countries to follow. Among the strategic actions of the Hepatitis C Action Plan for Scotland [50], a document was produced [51], with the aim to support NHS Boards build action plans for facilitating, delivering and evaluating workforce education development for staff. Complements this a workbook [52], published to provide staff with a structured approach to assessing, demonstrating and developing their ability to carry out their role in delivering Hepatitis C services. Scaling up HPs training and evaluating the compliance to clinical practice guidelines were also objectives of the French National Plan for hepatitis B and C 2009–2012, following which the management of hepatitis B and C was set as a priority topic in continuing medical education [53]. Subsequently, guidelines for the management of patients with hepatitis B and C were developed [54], with recommendations aimed at HPs and the other key stakeholders.

## Conclusions

Our results suggest that not only are there few examples of guidelines for the professional groups most able to implement the recommendations, but also that there is low awareness of those that do exist among primary care professionals most often representing patients' first points of contact. Without short, precise and feasible guidelines, there are likely to be wide inconsistencies in screening, referral and patient management. Results from our survey also suggest that scientific databases are not the most important information source of best clinical practice for many HPs. Implementation of best practices at both national and European level requires not only the availability of high quality guidelines tailored to the needs of the different professional groups, but also that their existence is actively promoted among those who are to follow them, especially when we consider that availability of research evidence alone does not necessarily coincide with the adoption of recommended practices by physicians. The availability of relevant summaries within guidelines, as well as target dissemination to less experienced clinicians, along with the provision of clear and concise information to patients, are possible solutions to enhance guidelines implementation among clinicians. Given the growing interest in knowledge translation and research dissemination, our findings could prompt key decision-making bodies to improve physicians’ awareness, agreement, adoption and adherence to clinical practice guidelines, for example through professional associations and training. Our results show that knowledge and availability of hepatitis B/C training could also be improved. Further studies assessing the impact of existing training and guidelines on the care, health literacy and onward referral of patients would be very valuable.

## Additional files

Additional file 1: Table S1. Hepatitis B/C related guidance documents retrieved by the literature search and/or identified by experts. (DOCX 36 kb) Additional file 2: Tables 2, 3, 4 and 6 with data presented in absolute numbers. Health professionals reporting the existence of national general hepatitis B and C guidelines in their country. **Table 3.** Health professionals identifying hepatitis general or specific guidelines by professional group and by country. **Table 4.** Availability of training to improve knowledge and skills in viral hepatitis in the six countries. **Table 6.** Health professionals’ opinion on the existence of barriers as explanations of why hepatitis B/C cases do not reach specialized health care (e.g. hepatologists) for further investigation and treatment. Results are presented by country. (DOCX 23 kb)

## Abbreviations

ANC: Antenatal care
ASC: Asylum seekers care
CDC: Centers for Disease Control and Prevention
DE: Germany
ES: Spain
EU: European Union
GP: General practitioner
GUM: Genito-Urinary Medicine
HBV: Hepatitis B virus
HCV: Hepatitis C virus
HP: Health Professional
HU: Hungary
IDU: Injecting Drug User
IT: Italy
NL: Netherlands
PHP: Public health professional
PWID: People who inject drugs
SHS: Sexual health services
UK: United Kingdom
