# Supplementary material for: Identification of hepatitis B and C screening and patient management guidelines and availability of training for chronic viral hepatitis among health professionals in six European countries: results of a semi-quantitative survey
Source: BMC Infect Dis. 2015 Aug 19;15:353. doi: 10.1186/s12879-015-1104-8 (PMC4545377; doi:10.1186/s12879-015-1104-8)
Supplement: Additional file 1: Table S1. — Hepatitis B/C related guidance documents retrieved by the literature search and/or identified by experts. (DOCX 36 kb) [file 12879_2015_1104_MOESM1_ESM.docx]

Table 2. Hepatitis B/C related guidance documents retrieved by the literature search and/or identified by experts.

| **Country** | **#** | **Produced by** | **Date** | **Title and URL** | **Identified/retrieved by** | |
| --- | --- | --- | --- | --- | --- | --- |
|  |  |  |  |  | **Experts via survey (specify)** | **The literature search** |
| **The United Kingdom** | 1 | Department of Health | Update 2013 | Immunisation against Infectious Disease. ‘The Green book’  <https://www.gov.uk/government/collections/immunisation-against-infectious-disease-the-green-book#the-green-book> | PHE, SHS |  |
|  | 2 | Department of Health | 2011 | Hepatitis B antenatal screening and newborn immunisation programme: Best practice guidance  <https://www.gov.uk/government/publications/hepatitis-b-antenatal-screening-and-newborn-immunisation-programme-best-practice-guidance> | PHE, AS |  |
|  | 3 | Department of Health | 2009 | Hepatitis C Quick reference guide for primary care  <http://www.nhs.uk/hepatitisc/SiteCollectionDocuments/pdf/Hepatitis_C_quick_reference_for_PC_April2011.pdf> | PHE, Specialist |  |
|  | 4 | HPA | 2011 | Standards for local surveillance and follow up of hepatitis B and C.  <http://www.hpa.org.uk/webc/hpawebfile/hpaweb_c/1194947376936> | PHE, SHS, ANC |  |
|  | 5 | UK National Screening Committee | Update 2012 | Infectious Diseases in Pregnancy Screening (IDPS) Programme. Programme Standards.  <http://infectiousdiseases.screening.nhs.uk/standards> | PHE, ANC, GP, AS |  |
|  | 6 | UK Screening Programme | 2011 | Antenatal Screening For Hepatitis C  <http://www.screening.nhs.uk/hepatitisc-pregnancy> | GP |  |
|  | 7 | Scottish Government | 2011 | The Sexual Health and Blood Borne Virus Framework 2011-2015  <http://www.scotland.gov.uk/Publications/2011/08/24085708/0> | SHS |  |
|  | 8 | NHS Scotland | 2006-08 | Hepatitis C Action Plan for Scotland  <http://www.scotland.gov.uk/Publications/2006/09/15093626/13> | GP, Specialist | X |
|  | 9 | NHS Lothian | 2011 | BBV Testing in Adults Guideline  <http://www.refhelp.scot.nhs.uk/dmdocuments/Infectious%20Diseases/BBV%20testing%20Guideline%202010%20NHS%20Lothian.pdf> | SHS |  |
|  | 10 | National Institute for Health and Care Excellence (NICE) | 2007 | TA 96 Adefovir dipivoxil and peginterferon alfa‐2a for the treatment of chronic hepatitis B. <http://guidance.nice.org.uk/TA96> | Specialist | X |
|  | 11 | NICE | 2009 | TA 153 Entecavir for the treatment of chronic hepatitis B.  <http://guidance.nice.org.uk/TA153> | Specialist |  |
|  | 12 | NICE | 2009 | TA 154 Telbivudine for the treatment of chronic hepatitis B.  <http://guidance.nice.org.uk/TA154> | Specialist |  |
|  | 13 | NICE | 2012 | TA 173 Tenofovir disoproxil fumarate for the treatment of hepatitis B.  <http://guidance.nice.org.uk/TA173> | Specialist |  |
|  | 14 | Scottish Intercollegiate Guidelines Network (SIGN) | 2013 | Management of hepatitis C: A national clinical guideline (SIGN 133 – update to SIGN 92 (2006)  <http://www.sign.ac.uk/pdf/sign133.pdf> | PHE | X |
|  | 15 | British Association for Sexual Health and HIV (BASHH) | 2008 | UK National Guideline on the Management of the Viral Hepatitides A, B & C  <http://www.bashh.org/documents/1927.pdf> | ANC, SHS |  |
|  | 16 | The Royal College of General Practitioners | 2007 | Guidance for the prevention, testing, treatment and management of hepatitis C in primary care  <http://www.rcgp.org.uk/revalidation-and-cpd/~/media/Files/SMAH/RCGP-Guidance-for-prevention-testing-treatment-and-management-of-hepatitis-C-in-primary-care-2007.ashx> | PHE |  |
|  | 17 | British Viral Hepatitis Group (BVHG) | 2008 | UK guidelines for the initial management of hepatitis B infection:  BVHG Consensus Statement ‘UK guidelines for the management of babies born to women who are HBsAg positive’  <http://infectiousdiseases.screening.nhs.uk/getdata.php?id=10844>. | ANC |  |
|  | 18 | British HIV Association (BHIVA) | Update 2013 | Guidelines for the management of hepatitis viruses in adults infected with HIV <http://www.bhiva.org/documents/Guidelines/Hepatitis/2013/HepatitisGuidelines2013.pdf> | ANC, SHS |  |
|  | 19 | British Society of Gastroenterology | 2001 | Clinical guidelines on the management of hepatitis C  <http://www.bsg.org.uk/clinical-guidelines/liver/clinical-guidelines-on-the-management-of-hepatitis-c.html> | Specialist | X |
|  | 20 | European Association for the Study of the liver (EASL) | 2012 | Management of chronic hepatitis B virus infection | Specialist | X |
|  | 21 | European Association for the Study of the liver (EASL) | 2011 | Management of hepatitis C virus infection | Specialist | X |
|  | 22 | NICE | 2004 | Interferon alfa (pegylated and non-pegylated) and ribavirin for the treatment of chronic hepatitis C  <http://publications.nice.org.uk/interferon-alfa-pegylated-and-non-pegylated-and-ribavirin-for-the-treatment-of-chronic-hepatitis-ta75> |  | X |
|  | 23 | NICE | Update  2014 | NICE public health guidance. Needle and syringe programmes <http://guidance.nice.org.uk/PH52> |  | X |
| **Germany** | 1 | Arbeitsgemeinschaft der Wissenschaftlichen Medizinischen Fachgesellschaften (AWMF) *(Association of the Scientific Medical Societies in Germany*) | 2011 | Hepatitis B Virusinfektion: Prophylaxe, Diagnostik und Therapie (S3‐Leitlinie, AWMF 021/011)  <http://www.awmf.org/leitlinien/detail/ll/021-011.html> | PHE, ANC, ASCP, SHS, Sp. | X |
|  | 2 | AWMF | 2009 | Hepatitis C Virus (HCV): Infektion, Prophylaxe, Diagnostik und Therapie (S3‐Leitlinie, AWMF 021/012)  <http://www.awmf.org/leitlinien/detail/ll/021-012.html> | All | X |
|  | 3 | RKI - Ratgeber für Ärzte | 2012 (update 2013) | Epid. Bull. 1/2012 (Hepatitis B)  <http://www.rki.de/DE/Content/Infekt/EpidBull/Merkblaetter/Ratgeber_HepatitisB.html> | PHE, ASCP, SHS |  |
|  | 4 | RKI - Ratgeber für Ärzte | 2012 (update 2014) | Epid. Bull. 4/2012 (Hepatitis C)  <http://www.rki.de/DE/Content/Infekt/EpidBull/Merkblaetter/Ratgeber_HepatitisC.html> | PHE, SHS |  |
|  | 5 | Nationale Stillkommission | 2008 | Hepatitis C und Stillen *(Hepatitis C and breast feeding - second updated recommendation of the national breast feeding commission 30. May 2008)* <http://www.bfr.bund.de/cm/343/hepatitis_c_und_stillen_zweite_ergaenzung.pdf> | ANC |  |
|  | 6 | Gemeinsamer Bundesausschuss | 2012 (update 2014) | Mutterschaftsrichtlinien, GBA *(Guidelines for physicians on care during the period of pregnancy and delivery)* <https://www.g-ba.de/informationen/richtlinien/19/> | ANC |  |
| **The Netherlands** | 1 | The International Union against Sexually Transmitted Infections (IUSTI) | 2010 | European guideline for the management of hepatitis B and C virus infections  <http://www.iusti.org/regions/europe/pdf/2010/Euro_guideline_Hep%20B_C%20Europe_04.10.pdf> | SHS | X |
|  | 2 | Rijksinstituut voor Volksgezondheid en Milieu (RIVM) *(The National Institute for Public Health and the Environment)* | HBV 2013 (update)  HCV 2011 | LCI-richtlijn Hepatitis B and C <http://www.rivm.nl/Documenten_en_publicaties/Professioneel_Praktisch/Richtlijnen/Infectieziekten/LCI_richtlijnen/LCI_richtlijn_Hepatitis_B> OR  <http://www.rivm.nl/Documenten_en_publicaties/Professioneel_Praktisch/Richtlijnen/Infectieziekten/LCI_richtlijnen/LCI_richtlijn_Hepatitis_C> | All |  |
|  | 3 | RIVM | 2011 | Bescherming tegen hepatitis B voor baby’s van dragermoeders  *(“CIb Protocol Hepatitis B immunisation of newborns of HBsAg positive mothers”)*  <http://www.rivm.nl/Documenten_en_publicaties/Professioneel_Praktisch/Protocollen/Infectieziekten/Rijksvaccinatieprogramma/Factsheet_Bescherming_tegen_hepatitis_B_voor_baby%E2%80%99s_van_dragermoeders> | PHE |  |
|  | 4 | RIVM | 2012 | Landelijke richtlijn preventie transmissie van hepatitis B van medisch personeel naar patiënten.  (*'National guideline prevention of hepatitis B virus transmission from health care workers to patients')*  <http://www.rivm.nl/dsresource?objectid=rivmp:53526&type=org&disposition=inline&ns_nc=1> | Specialist |  |
|  | 5 | Nederlands Huisartsen Genootschap (NHG) *(Dutch College of General Practitioners)* | 2007 | Virushepatitis en andere leveraandoeningen  (‘*Viral hepatitis and other liver diseases’*)  <https://www.nhg.org/standaarden/samenvatting/virushepatitis-en-andere-leveraandoeningen> | All |  |
|  | 6 | NV MDL *(Dutch Association for Specialists in Gastroenterology & Hepatology)* | 2012 (HBV) | Richtlijn behandeling van chronische hepatitis-B-virusinfectie  *(Treatment Guideline for chronic hepatitis B virus infection)*  <http://www.mdl.nl/uploads/240/1109/Richtlijn_HBV_nieuwe_inzichten_2012.pdf>  English version: <http://www.njmonline.nl/getpdf.php?t=a&id=10000884> | PHE, GP, Specialist | X |
|  | 7 | NV MDL *(Dutch Association for Specialists in Gastroenterology & Hepatology)* | 2011 | Richtlijn hepatitis C monoinfectie  *(Hepatitis C monoinfection guideline)* <http://www.mdl.nl/uploads/240/1336/Richtlijn_HCV_definitief_t.b.v_website.pdf>  English version: <http://www.njmonline.nl/getpdf.php?t=a&id=10001001> | PHE, GP, ANC, Specialist | X |
|  | 8 | CBO (Centraal BegeleidingsOrgaan) *(The Dutch Institute for health care improvement)* | 2002 | Seksueel overdraagbare aandoeningen en herpes neonatorum  (*Sexually transmitted diseases and neonatal herpes*)  <http://www.diliguide.nl/document/1268> | AS |  |
| **Hungary** | 1 | A Nemzeti Erőforrás Minisztérium  *(The Ministry of National Resources)* | 2011 | Szakmai protokollja a C hepatitis antivirális kezelésérő (Eü. Közlöny LXI. évf. 7. szám)  *(Professional protocol for addressing the viral hepatitis C (Medical Journal 2011. Vol. LXI No. 7 pg. 1393)*  <http://www.kozlonyok.hu/kozlonyok/Kozlonyok/6/PDF/2011/7.pdf> | PHE, AS |  |
|  | 2 | Országos Közegészségügyi Intézet (jogutódja OEK) (*National Institute of Public Health)* |  | Tájékoztató A HBsAg pozitív terhes nőknek  (*Memorandum for HBsAg positive pregnant women)* | PHE |  |
|  | 3 | Orvosi Hetilap (OH)  *(Journal of the Hungarian Medical Association)* | 2010, 151; 24-28 | Protokol a B‐és D hepatitis antivirális kezelésére  *(Protocol B and hepatitis C antiviral treatment)*  <http://www.akademiai.com/content/n66183h7266q7845/?p=fb7999b84fbc4a32bc7faebf18c4067b&pi=6> | AS |  |
|  | 4 | OH  *(Journal of the Hungarian Medical Association)* | 2012, 153: 375-394 | Ajánlás a B-, a C- és a D- vírus hepatitisek diagnosztikájára és kezelésére.  *(Recommendations for the diagnosis and treatment of viral hepatitis B-, C-and D)*  <http://www.akademiai.com/content/6263070941l24553/?p=120c87be9dea4e8b83c94c95e128144c&pi=1> | PHE, Specialist |  |
|  | 5 | The national consensus conference organized by specialist including:  - Magyar Gasztroenterológiai Társaság (MGT) *(Hungarian Society for Gastroenterology)*  - Magyar Infektológiai és Klinikai Mikrobiológiai Társaság *(Hungarian Society of Clinical Microbiology and Infectious Diseases)* | Updated annually | Ajánlása, évente frissítve a szakorvosokkal együtt rendezett országos konszenzus konferencián alapulva.  *(Annual update of recommendations from the national consensus conference organized by specialists)* | Specialist, AS, ANC |  |
|  | 6 | Hepatológiai szekció, Országos Egészségbiztosítási Pénztár (OEP) *(Hepatology Section, National Health Insurance Fund)* | 2010 | Krónikus B‐virus hepatitis kezelése  *(Treatment of chronic hepatitis B virus)*  <http://www.oep.hu/pls/portal/docs/PAGE/SZAKMA/OEPHUSZAK_EUSZOLG/FINANSZ%C3%8DROZ%C3%81SI%20PROTOKOLL/ARCH%C3%8DVUM/FINANSZ%C3%8DROZ%C3%81SI%20PROTOKOLLOK%20-%20ARCH%C3%8DVUM%202010.%20J%C3%9ANIUS/9.PDF> | Specialist |  |
|  | 7 | Lege Artis Medicinae Journal | 2006, 16(11) | Hepatitis C – Tények és dilemmák  *(Hepatitis C - Facts and dilemmas)*  <http://www.elitmed.hu/kiadvanyaink/lam_lege_artis_medicin/hepatitis_c_tenyek_es_dilemmak-528/> | AS |  |
| **Italy** | 1 | European Association for the Study of the liver (EASL) | 2012 | Management of chronic hepatitis B virus infection. | PHE, ANC, GP | X |
|  | 2 | European Association for the Study of the liver (EASL) | 2011 | Management of hepatitis C virus infection | GP | X |
|  | 3 | AASLD | 2009 | AASLD Practice Guidelines: Chronic Hepatitis B: Update 2009 <http://www.aasld.org/practiceguidelines/Documents/Bookmarked%20Practice%20Guidelines/Chronic_Hep_B_Update_2009%208_24_2009.pdf> | Specialist | X |
|  | 4 | Ministero della Salute, ISS, CeVEAS | 2011 | National GL for the management of the physiological pregnancy  *(Linea guida sulla gravidanza fisiologica)* | ANC |  |
|  | 5 | Ministry of Health | 2012-14 | National Prevention Plan (*Piano Nazionale della Prevenzione*) | ANC |  |
|  | 6 | Ministry of Health | 1991, 2000 | Ministerial Decrees 0/10/1991 and 20/11/2000 (HBV vaccination for at-risk categories) ( *Decreto ministeriale 3 ottobre 1991 and Decreto ministeriale20 novembre 2000)* | ANC |  |
|  | 7 | AISF (*Italian Association for the Study of the Liver*), SIMIT (*Italian Society of Infectious and Tropical Diseases*) FederSerD (*Italian Federation Department's Operators and Addiction Services*), *Italian Prison Medicine and Healthcare Society* (S.I.M.S.Pe.) | 2011 | Recommendations for the prevention, diagnosis, and treatment of chronic hepatitis B and C in special population groups (migrants, intravenous drug users and prison inmates)  <http://www.ncbi.nlm.nih.gov/pubmed/21256097> | ANC, Specialist |  |
|  | 8 | AISF, SIMIT, SIMAST (*Italian Society for the Study of Sexually Transmitted Diseases*) | 2010 | Practice guidelines for the treatment of hepatitis C: recommendations from an AISF/SIMIT/SIMAST Expert Opinion Meeting <http://www.ncbi.nlm.nih.gov/pubmed/19748329> | GP, SHS, Specialist | X |
|  | 9 | SNLG- Istituto Superiore di Sanità (*National guidelines system - National Institute of Health*) | 2006 | Lo screening per infezione da virus dell’epatite C negli adulti in Italia *(Screening for hepatitis C infections in adults)* <http://www.snlg-iss.it/cms/files/CC_epatite_C.pdf> | ANC, GP |  |
|  | 10 | Emilia‐ Romagna Region | 2010 | Treatment of adults with chronic hepatitis B (*Trattamento della epatite cronica B nell’adulto*) <http://www.saluter.it/documentazione/ptr/elaborati/103-linee-guida-epatite-cronica-b> | ANC |  |
|  | 11 | (Società Interdisciplinare per lo Studio delle Malattie Sessualmente  Trasmissibili, Società  Italiana di Gastroenterologia, Associazione Pazienti Epac Onlus, Associazione Italiana per lo Studio del  Fegato, (Società Italiana di Medicina Generale | 2010 | Epatiti: un’emergenza sommersa <http://www.sosfegato.it/camo/onlus/es/Documento_indirizzo.pdf> |  | X |
| **Spain** | 1 | AAEH (Asociación Española para el Estudio del Hígado). Published in Gastroenterol Hepatol. | 2012;  35(7): 512--528 | Documento de consenso de la AEEH sobre el tratamiento de la infección por el virus de la hepatitis B.  *(Consensus document of the on the treatment of Hepatitis B infection)*  <http://aeeh.es/wp-content/uploads/2012/09/Documento-de-consenso-VHB-de-la-AEEH.pdf> | Specialist |  |
|  | 2 | Asociación Española para el Estudio del Hígado (AEEH) | 2009 | Diagnóstico y tratamiento de las enfermedades hepáticas  *(Diagnosis and treatment of liver diseases)* | Specialist |  |
|  | 3 | Rev Esp Sanid Penit  *(Spanish Journal of Prison Health)* | 2009, 11: 87‐95 | Orientaciones para un mejor manejo de la hepatitis B en España  *(Guidance for better management of hepatitis B in Spain)*  <http://scielo.isciii.es/scielo.php?script=sci_arttext&pid=S1575-06202009000300005&lng=en&nrm=iso> | Specialist |  |
|  | 4 | Grupo de Prevención de Enfermedades Infecciosas del Programa de Actividades Preventivas y de Promoción de la Salud (PAPPS)  *(Group on Prevention of Infectious Diseases from Program of Preventive Activities and Health Promotion PAPPS)* | 2003 | Prevención de las enfermedades infecciosas  *(Prevention of infectious diseases)*  <http://www.papps.org/upload/file/publicaciones/manual/modulo%204.pdf> | PHE |  |
|  | 5 | European Association for the Study of the liver (EASL) | 2012 | Management of chronic hepatitis B virus infection. | Specialist | X |
|  | 6 | European Association for the Study of the liver (EASL) | 2011 | Management of hepatitis C virus infection | Specialist | X |
|  | 7 | AASLD | 2009 | AASLD Practice Guidelines: Chronic Hepatitis B: Update 2009 <http://www.aasld.org/practiceguidelines/Documents/Bookmarked%20Practice%20Guidelines/Chronic_Hep_B_Update_2009%208_24_2009.pdf> | Specialist | X |
| **International** | 1 | AASLD | **2009** | AASLD Practice Guidelines: Diagnosis, Management, and Treatment of Hepatitis C: Update  <http://onlinelibrary.wiley.com/doi/10.1002/hep.22759/pdf> |  | X |
|  | 2 | European Viral Hepatitis Educational Initiative (EVHEI) | **2007** | Best practice in the treatment of chronic hepatitis B: a summary of the European Viral Hepatitis Educational Initiative (EVHEI)  [http://www.sciencedirect.com/science/article/pii/S0168827807004138#](http://www.sciencedirect.com/science/article/pii/S0168827807004138) |  | X |
|  | 3 | NIH | **2002** | National Institutes of Health Consensus Conference Statement. Management of Hepatitis C  <http://consensus.nih.gov/2002/2002hepatitisc2002116html.htm> |  | X |
|  | 4 | NIH | **2008** | National Institutes of Health Consensus Conference Statement.  Management of Hepatitis B <http://consensus.nih.gov/2008/hepb.htm> |  | X |

PHE= Public Health Experts; GPs= general practitioners; SHS=sexual health service providers and/or genitourinary medicine specialists; ANC= antenatal care providers; ASC=asylum seeker care providers; specialist= specilaists in the field of gastroenterology/hepatology and infectious diseases.
