# Supplementary material for: Identification of hepatitis B and C screening and patient management guidelines and availability of training for chronic viral hepatitis among health professionals in six European countries: results of a semi-quantitative survey
Source: BMC Infect Dis. 2015 Aug 19;15:353. doi: 10.1186/s12879-015-1104-8 (PMC4545377; doi:10.1186/s12879-015-1104-8)
Supplement: Additional file 2: Tables 2, 3, 4 and 6 with data presented in absolute numbers. — Health professionals reporting the existence of national general hepatitis B and C guidelines in their country. Table 3. Health professionals identifying hepatitis general or specific guidelines by professional group and by country. Table 4. Availability of training to improve knowledge and skills in viral hepatitis in the six countries. Table 6. Health professionals’ opinion on the existence of barriers as explanations of why hepatitis B/C cases do not reach specialized health care (e.g. hepatologists) for further investigation and treatment. Results are presented by country. (DOCX 23 kb) [file 12879_2015_1104_MOESM2_ESM.docx]

**Additional file 2**

**Table 2. Health professionals reporting the existence of national general hepatitis B and C guidelines in their country.**

| **Hepatitis B guidelines** | **UK (n=47)** | **DE (n=64)** | **NL (n=55)** | **HU (n=21)** | **IT (n=58)** | **ES (n=23)** | **Total (n=268)** |
| --- | --- | --- | --- | --- | --- | --- | --- |
| Proportion of health professionals reporting the existence | 27 | 36 | 43 | 14 | 33 | 10 | 163 |
| Provided name and publisher * | 11 | 11 | 19 | 5 | 15 | 5 | 66 |
| **Hepatitis C guidelines** | **UK (n=47)** | **DE (n=64)** | **NL (n=55)** | **HU (n=21)** | **IT (n=58)** | **ES (n=23)** | **Total (n=268)** |
| Proportion of health professionals reporting the existence | 28 | 30 | 37 | 14 | 33 | 9 | 151 |
| Provided name and publisher ** | 11 | 11 | 19 | 4 | 15 | 4 | 64 |

* % of the respondents who reported the existence of general hepatitis B guidelines

** % of the respondents who reported the existence of general hepatitis C guidelines

**Table 3. Health professionals identifying hepatitis general or specific guidelines by professional group and by country.**

| **HBV GUIDELINES** | **UK** | **DE** | **NL** | **IT** | **ES** | **HU** | Total |
| --- | --- | --- | --- | --- | --- | --- | --- |
| **General Hepatitis B guidelines** | | | | | | | |
| **Public health professionals** | 5 | 10 | 5 | 4 | 4 | 2 | 30 |
| **ANC experts** | 4 | 11 | 4 | 10 | 1 | 2 | 32 |
| **General Practitioners** | 3 | 2 | 7 | 10 | 2 | 1 | 25 |
| **Asylum seekers Experts** | 4 | 3 | 2 | 3 | 0 | 3 | 15 |
| **SHS Experts** | 5 | 3 | 6 | 1 | 0 | 2 | 17 |
| **Specialists** | 6 | 7 | 19 | 5 | 3 | 4 | 44 |
| **GP guidelines** | | | | | | | |
| **Public health professionals** | 6 | 4 | 7 | 2 | 2 | 0 | 21 |
| **General Practitioners** | 1 | 0 | 7 | 3 | 0 | 0 | 11 |
| **Antenatal guidelines** | | | | | | | |
| **Public health professionals** | 7 | 1 | 5 | 2 | 4 | 1 | 20 |
| **Antenatal care Experts** | 6 | 13 | 4 | 9 | 7 | 4 | 43 |
| **Asylum seekers guidelines** | | | | | | | |
| **Public health professionals** | 3 | 0 | 1 | 1 | 0 | 0 | 5 |
| **Asylum seekers Experts** | 0 | 0 | 0 | 0 | 0 | 0 | 0 |
| **Specialists guidelines** | | | | | | | |
| **Public health professionals** | 7 | 5 | 4 | 2 | 2 | 1 | 21 |
| **Specialists** | 4 | 3 | 19 | 3 | 3 | 4 | 36 |
| **Specific migrant care guidelines** | | | | | | | |
| **Public health professionals reporting the**  **existence of specific migrant care GLs** | 3 | 0 | 1 | 2 | 4 | 0 | 10 |
| **HCV GUIDELINES** | **UK** | **DE** | **NL** | **IT** | **ES** | **HU** | Total |
| **General Hepatitis C guidelines** | | | | | | | |
| **Public health professionals** | 5 | 10 | 4 | 4 | 4 | 2 | 29 |
| **ANC experts** | 3 | 6 | 4 | 10 | 2 | 1 | 26 |
| **General Practitioners** | 3 | 2 | 6 | 10 | 1 | 1 | 23 |
| **Asylum seekers Experts** | 4 | 3 | 2 | 3 | 0 | 3 | 15 |
| **SHS Experts** | 6 | 3 | 5 | 1 | 0 | 2 | 17 |
| **Specialists** | 7 | 6 | 16 | 5 | 2 | 5 | 41 |
| **GP guidelines** | | | | | | | |
| **Public health professionals** | 6 | 3 | 4 | 3 | 2 | 0 | 18 |
| **General Practitioners** | 0 | 0 | 5 | 3 | 0 | 0 | 8 |
| **Antenatal guidelines** | | | | | | | |
| **Public health professionals** | 3 | 0 | 1 | 2 | 0 | 0 | 6 |
| **Antenatal care experts** | 2 | 4 | 0 | 8 | 6 | 1 | 21 |
| **Asylum seekers guidelines** | | | | | | | |
| **Public health professionals** | 1 | 0 | 0 | 1 | 0 | 0 | 2 |
| **Asylum seekers Experts** | 0 | 0 | 0 | 0 | 0 | 0 | 0 |
| **Specialists guidelines** | | | | | | | |
| **Public health professionals** | 5 | 3 | 2 | 2 | 0 | 0 | 12 |
| **Specialists** | 4 | 3 | 16 | 4 | 2 | 4 | 33 |
| **Specific migrant guidelines** | | | | | | | |
| **Public health professionals reporting the**  **existence of specific migrant care GLs** | 3 | 0 | 0 | 1 | 2 | 0 | 6 |

GLs: Guidelines

**Table 4. Availability of training to improve knowledge and skills in viral hepatitis in the six countries.**

| **UK** | **GP (n=10)** | **Antenatal (n=8)** | **Asylum (n=4)** | **SHS (n=10)** | **Specialist (n=10)** |
| --- | --- | --- | --- | --- | --- |
| Yes | 4 | 4 | 1 | 7 | 10 |
| No | 1 | 1 | 3 | 0 | 0 |
| Unsure | 5 | 3 | 0 | 3 | 0 |
| **DE** | **GP (n=4)** | **Antenatal (n=36)** | **Asylum (n=3)** | **SHS (n=5)** | **Specialist (n=9)** |
| Yes | 2 | 4 | 2 | 3 | 6 |
| No | 0 | 9 | 1 | 0 | 0 |
| Unsure | 2 | 23 | 0 | 2 | 3 |
| **NL** | **GP (n=9)** | **Antenatal (n=6)** | **Asylum (n=4)** | **SHS (n=8)** | **Specialist (n=22)** |
| Yes | 8 | 2 | 3 | 5 | 22 |
| No | 0 | 3 | 1 | 1 | 0 |
| Unsure | 1 | 1 | 0 | 2 | 0 |
| **HU** | **GP (n=1)** | **Antenatal (n=4)** | **Asylum (n=3)** | **SHS (n=3)** | **Specialist (n=10)** |
| Yes | 1 | 2 | 1 | 0 | 8 |
| No | 0 | 1 | 1 | 3 | 0 |
| Unsure | 0 | 1 | 1 | 0 | 2 |
| **IT** | **GP (n=14)** | **Antenatal (n=25)** | **Asylum (n=3)** | **SHS (n=1)** | **Specialist (n=9)** |
| Yes | 11 | 7 | 0 | 0 | 4 |
| No | 1 | 13 | 3 | 1 | 3 |
| Unsure | 2 | 5 | 0 | 0 | 2 |
| **ES** | **GP (n=2)** | **Antenatal (n=8)** | **Asylum (n=1)** | **SHS (n=2)** | **Specialist (n=4)** |
| Yes | 1 | 6 | 0 | 1 | 4 |
| No | 1 | 2 | 1 | 0 | 0 |
| Unsure | 0 | 0 | 0 | 1 | 0 |

**Table 6. Health professionals’ opinion on the existence of barriers as explanations of why hepatitis B/C cases do not reach specialized health care (e.g. hepatologists) for further investigation and treatment. Results are presented by country.**

|  | | **UK (n=47)** | **DE (n=64)** | **NL (n=55)** | **HU (n=21)** | **IT (n=58)** | **ES (n=23)** |
| --- | --- | --- | --- | --- | --- | --- | --- |
| **There is limited guidance available to primary health care professionals about onward referral, counselling and patient management of hepatitis B/C patients.** | **Strongly disagree** | 5 | 5 | 1 | 7 | 4 | 4 |
|  | **Disagree** | 15 | 20 | 32 | 6 | 20 | 9 |
|  | **Neither agree or disagree** | 14 | 24 | 10 | 7 | 9 | 3 |
|  | **Agree** | 10 | 13 | 10 | 1 | 23 | 7 |
|  | **Strongly Agree** | 3 | 2 | 2 | 0 | 2 | 0 |
| **Although training on viral hepatitis management is available for health care providers, uptake is generally low among professionals.** | **Strongly disagree** | 1 | 5 | 1 | 6 | 2 | 3 |
|  | **Disagree** | 11 | 20 | 5 | 5 | 12 | 10 |
|  | **Neither agree or disagree** | 17 | 29 | 31 | 7 | 13 | 8 |
|  | **Agree** | 16 | 10 | 17 | 2 | 30 | 2 |
|  | **Strongly Agree** | 2 | 0 | 1 | 1 | 1 | 0 |
